# Temperature affects predation of schistosome-competent snails by a novel invader, the marbled crayfish *Procambarus virginalis*

**DOI:** 10.1371/journal.pone.0290615

**Published:** 2023-09-13

**Authors:** Sara M. Faiad, Maureen A. Williams, Maurice Goodman, Susanne Sokolow, Julian D. Olden, Kaitlyn Mitchell, Ranja Andriantsoa, Julia Patricia Gordon Jones, Luciano Andriamaro, Pascaline Ravoniarimbinina, Jeanne Rasamy, Tsilavina Ravelomanana, Salohy Ravelotafita, Ranaivosolo Ravo, Peter Rabinowitz, Giulio A. De Leo, Chelsea L. Wood

**Affiliations:** 1 School of Aquatic and Fishery Sciences, University of Washington, Seattle, WA, United States of America; 2 Department of Biology, McDaniel College, Westminster, MD, United States of America; 3 Hopkins Marine Station, Dept. of Oceans and of Earth System Science, Doerr School of Sustainability, Stanford University, Stanford, CA, United States of America; 4 Marine Science Institute, University of California, Santa Barbara, Santa Barbara, CA, United States of America; 5 Réseau International Schistosomiase Environnement Aménagement et Lutte (RISEAL) Madagascar, Madagascar; 6 School of Natural Science, Bangor University, Bangor, Gwynedd, United Kingdom; 7 Department of Zoology and Animal Biodiversity, University of Antananarivo, Antananarivo, Madagascar; 8 Department of Environmental/Occupational Health Sciences, Global Health, University of Washington, Seattle, WA, United States of America; 9 Center for One Health Research (COHR), University of Washington, Seattle, WA, United States of America; 10 Woods Institute for the Environment, Stanford University, Stanford, CA, United States of America; Universita del Salento, ITALY

## Abstract

The human burden of environmentally transmitted infectious diseases can depend strongly on ecological factors, including the presence or absence of natural enemies. The marbled crayfish (*Procambarus virginalis*) is a novel invasive species that can tolerate a wide range of ecological conditions and colonize diverse habitats. Marbled crayfish first appeared in Madagascar in 2005 and quickly spread across the country, overlapping with the distribution of freshwater snails that serve as the intermediate host of schistosomiasis–a parasitic disease of poverty with human prevalence ranging up to 94% in Madagascar. It has been hypothesized that the marbled crayfish may serve as a predator of schistosome-competent snails in areas where native predators cannot and yet no systematic study to date has been conducted to estimate its predation rate on snails. Here, we experimentally assessed marbled crayfish consumption of uninfected and infected schistosome-competent snails (*Biomphalaria glabrata* and *Bulinus truncatus*) across a range of temperatures, reflective of the habitat range of the marbled crayfish in Madagascar. We found that the relationship between crayfish consumption and temperature is unimodal with a peak at ~27.5°C. Per-capita consumption increased with body size and was not affected either by snail species or their infectious status. We detected a possible satiation effect, i.e., a small but significant reduction in per-capita consumption rate over the 72-hour duration of the predation experiment. Our results suggest that ecological parameters, such as temperature and crayfish weight, influence rates of consumption and, in turn, the potential impact of the marbled crayfish invasion on snail host populations.

## Introduction

The human burden of environmentally transmitted infectious diseases depends strongly on ecological factors [1], such as the presence of natural enemies [2]. As antagonistic interactors that regulate the transmission of infectious disease, natural enemies can potentially include predators, competitors, or parasites of reservoir hosts or of the disease agent itself [2, 3]. However, evidence for disease transmission regulation by natural enemies is sparse, and generalities regarding the influence of these antagonists remain elusive [4, 5]. This knowledge gap is of particular concern, not just for understanding the potential role of natural enemies in controlling and eliminating infectious disease, but also because non-native species are becoming established in new regions at unprecedented rates [6]. Non-native species have long been recognized as having profound effects on ecosystem services [7–9]. Like native enemies, invasive species influence the abundance and distribution of their prey and competitors [10, 11], with important implications for transmission of environmentally transmitted infectious diseases–a phenomenon that has remained largely unexplored.

One such invader, with a rapidly expanding range and the potential to influence disease transmission, is the parthenogenetic marbled crayfish (*Procambarus virginalis*)—the only clonal decapod ever described [12]. The marbled crayfish is believed to have arisen from a non-clonal ancestor in the American Cambaridae family and is phylogenetically similar to *Procambarus fallax* [12]. First appearing in the German aquarium trade in the mid-1990s, this novel invader quickly spread across Europe [13]. Following its introduction to Madagascar in 2005, the marbled crayfish began to spread across the country [13–15], overlapping with the distribution of freshwater snails (*Biomphalaria pfeifferi* and *Bulinus* spp.) that serve as the intermediate hosts of schistosomiasis (*Schistosoma mansoni* and *S*. *haematobium*, respectively [16]).

Schistosomiasis is a parasitic disease endemic to tropical and subtropical regions [17]. Freshwater snails (largely from three genera: *Biomphalaria*, *Bulinus*, and *Oncomelania*) [18] play an obligate role in the transmission of *Schistosoma* spp. worms. Snails serve as intermediate hosts in the *Schistosoma* life cycle, incubating and shedding infectious *Schistosoma* cercariae (free-swimming, larval worms) into streams, ponds, and lakes. These cercariae go on to penetrate the skin of humans bathing, wading, washing laundry, or otherwise in contact with contaminated freshwaters. Over 250 million people are infected with schistosomiasis globally, totaling to 3.31 million disability-adjusted life years annually [19]. Though schistosomiasis infections rarely result in mortality, the disabilities associated with the disease are severe and burdensome, falling second in both disability-associated burden and prevalence only to intestinal nematode infections [19]. Notably, these estimates do not consider the economic and social impacts of disease, though it is widely understood that the compromised health of community members–a result of untreated schistosomiasis infections–reduces the opportunities for social and economic development [20–22]. Despite its impacts on human morbidity and mortality, schistosomiasis remains prevalent, disproportionately impacting poor and marginalized communities, particularly those that lack the public infrastructure needed for access to safe drinking water, adequate sanitation, and healthcare [23].

The World Health Organization (WHO) estimated that in 2019 over 235 million people were at risk of schistosomiasis infection and required preventative treatment, with 90% of those individuals living in Africa [24]. The standard treatment–an oral dose of the anthelmintic praziquantel–is considered effective in treating adult parasites (although there is rising concern of selection for resistance [25]); however, at this time, fewer than half of those in need of treatment (105,420,110 / 235,378,761 = 44.8%) receive it [24]. Mass drug administration has also fallen short due to high rates of re-infection, as individuals are often repeatedly exposed to contaminated water sources [26–30] and treatment is ineffective against juvenile worms [31–33]. In response, the WHO recognizes snail control as essential to reducing *Schistosoma* spp. transmission and recommends the implementation of snail control strategies, in combination with mass drug administration, to control and eliminate schistosomiasis [34]. Historically, snail control has been accomplished with chemical molluscicides and habitat modification, such as vegetation removal, draining wetlands, cementing canals, and alterations to water flow [35]. Alternatively, snail populations can be reduced by abundant populations of predators (e.g., crustaceans, birds, and fish) or competitors (e.g., other snail species). Indeed, natural enemies show some promise in the reduction of human schistosomiasis [36–39]. For example, the abundance of molluscivorous cichlids (*Trematocranus placodon*) was negatively correlated with intermediate snail host abundance, and temporal declines in fish abundance were associated with increases in schistosomiasis [39]. In the Senegal River Basin–the epicenter of the world’s largest intestinal schistosomiasis epidemic–areas previously inhabited by and now hypothesized to be absent of native river prawns experienced greater increases in schistosomiasis infections compared to regions falling outside of the prawns’ range [40], and localized reintroductions significantly reduced snail population numbers and, in turn, human disease prevalence [38].

While some introductions of non-native enemies in previous disease control efforts have resulted in unanticipated negative consequences [2], introduced species have also been used with success to reduce the abundance of zoonotic reservoirs of disease and of human disease burden [10, 41, 42]–representing a potential benefit of non-native species [43]. Regarding schistosomiasis, the abundance of the invasive red swamp crayfish (*Procambarus clarkii*) was significantly correlated with the disappearance of snails in Egyptian irrigation channels [41], and an established population of *P*. *clarkii* reduced snail abundance in Kenya [42]. Local schoolchildren were significantly less likely to become infected with *Schistosoma haematobium* where crayfish were present than in their absence–though environmental conditions influenced the crayfish’s impact on snail populations and local schistosomiasis prevalence and intensity [42].

Prey–predator relationships between gastropods and freshwater crayfish—both native and non-native—are well-documented [44], and numerous field and experimental studies highlight the regulatory impact of crayfish on snail populations [45–51]. Regulation of gastropod abundances by freshwater crayfish occurs through a combination of consumptive and nonconsumptive mechanisms, though it remains unclear which mechanism drives this widespread pattern [45, 52]. Snail populations decline in the presence of crayfish as a function of predation (i.e., a consumptive effect). On the other hand, the presence of predators, including crayfish, can indirectly impact snail populations if their presence results in changes in snail physiology (e.g., growth rate) [53–56], morphology [e.g., shell thickness, 57; shape, reviewed in 44], or behavior [e.g., habitat use, 54, 58, 59; feeding rate, 54] that, in some cases, decrease fitness [57]. Snail anti-predator behaviors include change in habitat use, reduced feeding rate, crawling up onto vertical substrates, and moving above the waterline (also known as “water quitting”)—all of which have been observed when snails are exposed to a crustacean predator [54–56, 59; additional behavioral responses to predators reviewed in 44].

Snails exposed to trematode infections, including *Schistosoma* spp., exhibit behavioral differences compared to uninfected conspecifics, which may alter predator risk and the rate at which snails are consumed by a predator [60–63]. Snails exposed to and presumably infected by *Schistosoma* move more slowly and less frequently than do uninfected conspecifics, suggesting that infected snails should be at greater risk for predation [60]. When exposed to simulated predation cues—a caged riverine prawn, *Macrobrachium vollenhovenii*, paired with crushed snail conspecifics—infected snails also showed a diminished anti-predator response compared to uninfected snails (where the anti-predator response is defined as “water quitting,” along with an aversion to open water and a preference for hiding under sheltered areas within the tank) [60]. Indeed, prawns preferentially consume *Bi*. *glabrata* snails exposed to *S*. *mansoni* and *Bu*. *truncatus* snails exposed to *S*. *haematobium* [60]. Differences in the consumption rate of uninfected and infected snails could alter the regulatory impact of crayfish on snail populations and, perhaps, human disease burden. Schistosomiasis control efforts may be enhanced by snail predators, if infected snails are consumed preferentially; however, intervention efforts may be hindered should predators avoid consuming infected snail hosts [60].

Like most crayfish [reviewed in 44], marbled crayfish are omnivores, said to eat “almost anything” [64]. However, plant material and snails seem to be their preferred food items [64]. Indeed, Andriantsoa et al. [65] anecdotally observed that native snails were absent from sites inhabited by this invader, suggesting that predation was not only occurring but that crayfish presence might reduce snail abundance to zero. Predation was later confirmed in a laboratory setting [65], highlighting the species’ potential to serve as a biological control of snails in Madagascar–something that is urgently needed in a country where prevalence in some villages can range up to 94% [66, 67].

The traits that make the marbled crayfish a particularly successful (and worrisome) invader may also make the species a formidable predator of Madagascar’s native snails, including schistosome-competent snails. Madagascar is diverse in habitat and climate [65]. Marbled crayfish can tolerate a wide range of ecological conditions and colonize diverse habitats [65], including areas outside of the limited geographic range of native crayfish species (Parastacidae; *Astacoides*) [68, 69]. The invader has been found in rice fields irrigated by thermal water reaching temperatures as high as 37°C, in 20°C river habitats [65] and has withstood temperatures as low as 5°C in a laboratory setting, though survivorship drastically declines at this extreme temperature [70]. Not only is this thermal plasticity and habitat diversity a feat for a clonal species, it also suggests that the marbled crayfish may be able to serve as a snail predator in habitats where other, endemically occurring snail predators cannot occur–perhaps, a collateral benefit of an otherwise destructive invasive species. However, rates of crayfish mortality, growth, and consumption vary greatly across temperatures [70–72]. Rates generally increase simultaneously with temperature until a thermal optimum is reached, beyond which point performance declines [73]. The temperature at which this thermal limit occurs varies considerably among crayfish species [74–76]. Marbled crayfish consumption (of carrots and worms [*Tubifex tubifex*]) ceases below 10°C [70], while the upper thermal limit remains unknown. Likewise, the thermal optimum of consumption, or the temperature at which consumption peaks, also remains unknown for marbled crayfish. As such, temperature plays a critical role in our understanding of the marbled crayfish’s potential as a biological control agent of snail intermediate hosts in Madagascar, as well as other regions in which the marbled crayfish has invaded and schistosomiasis is endemic.

This study identifies the conditions under which marbled crayfish prey on schistosome-competent snails. We addressed the following questions: i) Does temperature affect crayfish feeding rates on schistosome-competent snails and, if yes, is the relationship between temperature and feeding rate increasing, decreasing, or unimodal with a peak at intermediate temperatures? ii) Does the rate of crayfish consumption differ between snail species and between infected and uninfected snails across temperatures?, and iii) Does crayfish body size mediate the response of feeding rate to temperature?

We experimentally assessed marbled crayfish consumption of uninfected versus infected snails across a range of temperatures, reflective of the environmental conditions across the habitat range of the marbled crayfish in Madagascar. We hypothesized that the relationship between temperature and feeding rate would be unimodal with a peak at intermediate temperatures. Additionally, we hypothesized that snail infection status would influence the rate of consumption, as behavioral and physiological differences between uninfected and infected snails may make animals of differing infection status more or less accessible, detectable, or desirable to predators. Finally, we hypothesized that temperature may mediate the influence of weight on consumption, because temperature influences large-bodied organisms differently than those that are smaller in size [77]. Overall, the results from the present study enhance our understanding of the biotic and abiotic factors that impact the rate at which a recent invader, the marbled crayfish (*Procambarus virginalis*), consume schistosome-competent snails.

## Materials & methods

### Animal husbandry

Marbled crayfish were reared in freshwater aquaria filled with artificial pond water [78]. Crayfish tanks varied in size (3.72 L, 11.7 L, or 81.3 L), depending on the age, size, and rearing density of the crayfish. Crayfish, prior to becoming subjects in experiments, were typically housed with between two and four conspecifics. Juvenile crayfish were regularly removed from adult husbandry tanks and either relocated to a smaller tank (11.7 L) without adults or euthanized. Once included in the experiment, crayfish were housed individually in 11.7-L tanks. Husbandry tanks were held at room temperature (~25°C), whereas the temperature of experimental tanks was controlled and monitored (see below). All crayfish were regularly fed frozen carrots, except during experimental trials. Marbled crayfish (*Procambarus virginalis)* were obtained through private sellers on Etsy (https://www.etsy.com/) and Aquabid (https://www.aquabid.com/). Permission to import and house marbled crayfish for use in this study was provided by the State of Washington’s Department of Fish and Wildlife (Shellfish Import Permit No. 22–3020).

Snails were reared in freshwater aquaria (either 3.72- or 11.7-L tanks, depending on the density of snails), filled with artificial pond water [78]. Tanks underwent 100% water changes one to two times per week [79]. Snails were regularly fed romaine lettuce, which was refreshed during the bi-weekly water changes. All *Biomphalaria glabrata* (M-line, naive and exposed to *S*.*mansoni* strain PR-1) and *Bulinus truncatus* (Egypt, naive and exposed *S*. *haematobium* strain Egyptian) snails were provided by the NIAID Schistosomiasis Resource Center of the Biomedical Research Institute (Rockville, MD, USA) through NIH-NIAID Contract HHSN272201700014I for distribution through BRI Resources.

### Experiments

Our methods largely replicated previous experimental predation trials between crustaceans and *Schistosoma*-competent mollusks [80]. Briefly, one marbled crayfish (*Procambarus virginalis*) was held in combination with a set density (n = 12) of either *Bi*. *glabrata* or *Bu*. *truncatus* snails in a 11.7-L tank. An "average" size class of snails (6–10 mm shell length for *Bi*. *glabrata*; 5–10 mm shell length for *Bu*. *truncatus*) was used. Crayfish length and weight were measured prior to the start of each experimental period. Crayfish varied in weight between 1.54 g and 14.44 g with an average ± SE of 6.62 ± 0.117 g.

The total duration of each experimental period was 72 hours, with observations and snail replacement taking place every 12 hours. Each experimental period consisted of 7 total time points (0, 12, 24, 36, 48, 60, 72), in which each 12-hour increment constituted a “trial,” for a total of 6 trials per experimental period. At the conclusion of each trial, the number of snails above the water line, the number of snails on the lettuce (described below), the number of snails inside and under the shelter/hiding, and the number of snails in open water were counted and summed to reflect the total number of snails remaining in the tank. Additionally, the total number of empty, intact shells and the total number of dead snails were recorded at the conclusion of each trial. Shattered shell pieces were not included in empty shell counts, as it was too difficult to determine how many broken pieces constituted a singular shell. For each trial, we derived the total number of snails missing and presumably consumed as follows: the initial number of snails at each trial (*n* = 12), minus the number of snails remaining, minus the number of snails dead but not consumed. The number of snails consumed and the number of dead snails were totaled to determine the total number of snails to be replaced/added to the experimental tank. All counts were repeated and confirmed by a second observer. At the conclusion of each trial, dead snails and empty, intact shells were removed, snail density (*n* = 12) was reset, and the number of snails replaced/added was recorded. At the conclusion of the 72-hour experimental period, any remaining snails were removed from experiment tanks and returned to temperature-acclimated holding tanks. Crayfish remained in their tanks, allowing us to control for individual crayfish identity in analyses.

Crayfish and snails were provided with food throughout the duration of the experimental period. Specifically, at the beginning of the experimental period (time point “0”), a piece of romaine lettuce was added to each experimental tank to serve as a food source for snails. Additionally, one invertebrate pellet was placed into each of the tanks, including control tanks, to serve as an alternative source of food for crayfish. This reflected our assumption that crayfish are omnivorous, and are not limited to eating only snails in their natural habitats. Each experimental tank also contained a piece of PVC pipe, which served as a shelter for the crayfish and snails.

We observed crayfish consumption rate across five temperature conditions– 15, 20, 25, 30, and 35°C. This range reflects the diverse temperatures at which marbled crayfish have been found in Madagascar (20°C to 37°C) [65]. Though marbled crayfish can survive in temperatures as low as 5°C for extended periods of time, previous experiments have suggested that consumption ceases below 10°C [70]. However, consumption has been observed at 15°C, and therefore, this may reflect the lower thermal limit of crayfish feeding behavior. Animals underwent a temperature acclimation period, in which the water temperature changed 1–1.5°C/day until the desired temperature was reached. Animals were then held at the experimental temperature for at least 12 hours prior to the start of the experiment. Given that 15°C and 35°C would near the thermal limits of both the crayfish and snails [60, 65, 70, 81, 82], we included control tanks, from which crayfish were absent, to exclude the effect of temperature-associated snail death and ensure that snail mortality accurately reflected crayfish consumption.

We were interested in the influence of snail infection status on crayfish consumption rates and, therefore, varied snail infection status between experimental tanks. Each individual crayfish was held either with all “exposed” or “naive” (hereafter, “uninfected”) snails of one of two species included in the present study: *Biomphalaria glabrata* and *Bulinus truncatus*. Exposed snails were held at room temperature (~25°C) for ~30 days post-exposure (exposure date provided by the reagent provider, BRI) to allow infections to adequately mature [83] before being used in experiments. Following the post-exposure period, exposed snails were assumed to be infected and will be referred to as such hereafter.

**Fig 1:**
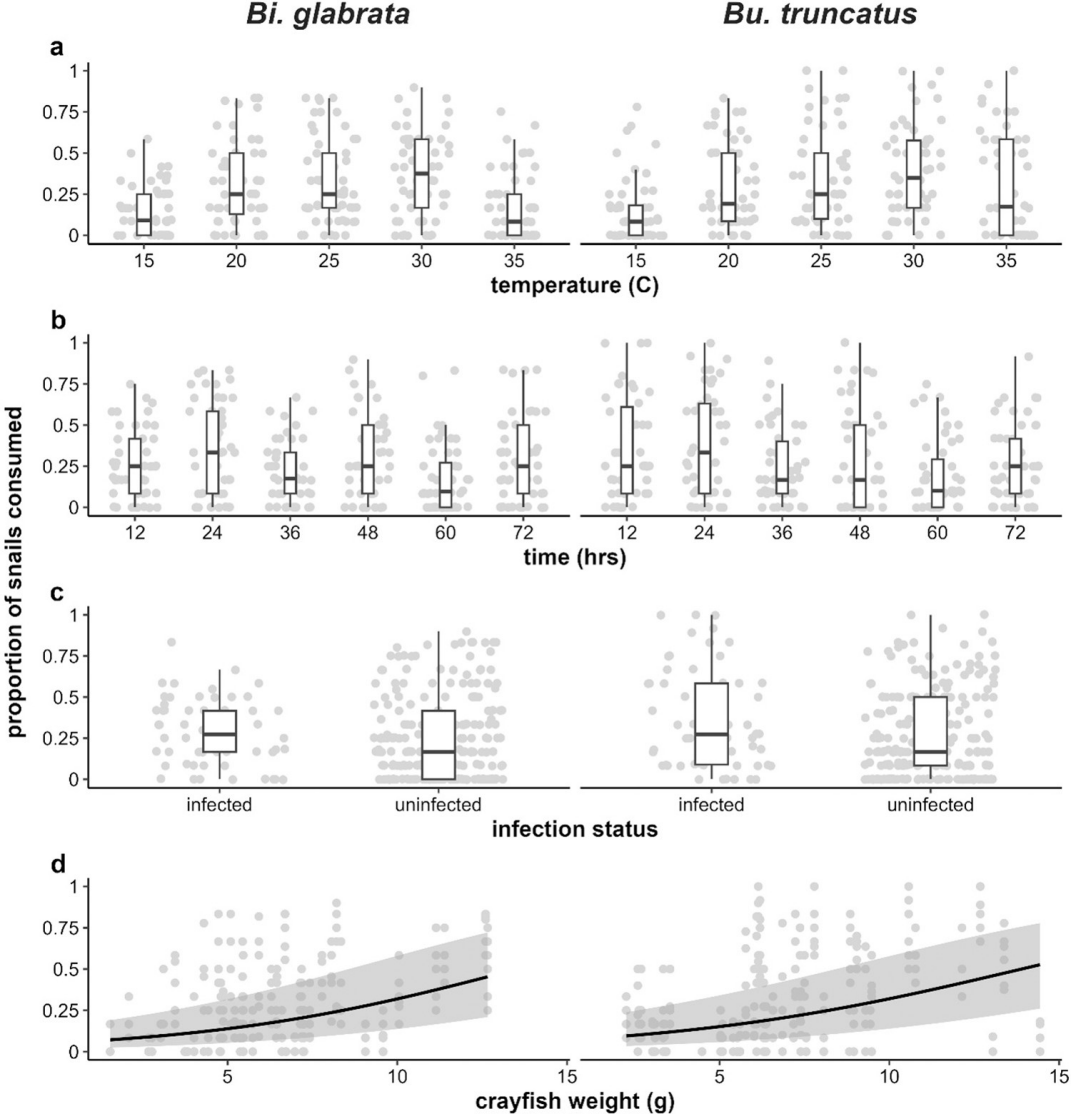
Bivariate plots displaying the proportion of snails consumed in each trial as jittered points with (a-c) overlayed boxplots for treatment variables, and (d) an overlayed regression curve (± standard error) for crayfish weight. Columns display data for each snail species, with rows displaying consumption across (a) temperature, (b) time block, (c) snail infection status, and (d) crayfish weight.

We were limited by the availability of infected *Bu*. *truncatus* and *Bi*. *glabrata* snails (Table 1, Fig 1). As such, the first round of experiments included only uninfected snails. Round 1 of experiments began on 14 June 2021 and concluded on 13 August 2021. In Round 1 of experiments, we conducted seven (72-hour long) predation experiments for each set temperature (15, 20, 25, 30, and 35°C) for a total of 42 (12-hour long) trials (observations) per temperature, with one crayfish individual held in combination with either uninfected *Bi*. *glabrata* or uninfected *Bu*. *truncatus* snails. Round 1 also included two (72-hour long) experiments in control tanks with snails and no crayfish for each set temperature for uninfected *Bi*. *glabrata* and *Bu*. *truncatus*, for a total of 14 (12-hour long) trials for each temperature for each species in control conditions Round 2 of experiments, which included both uninfected and infected snails, began on 25 October 2021 and concluded on 17 December 2021. In Round 2 of experiments, two 72-hour experiments, for a total of 14 (12-hour long) trials for each set temperature (15, 20, 25, 30, and 35°C) were conducted in both the experimental and control conditions for both uninfected and infected *Bi*. *glabrata* and *Bu*. *truncatus* snails. Crayfish individuals used in Round 1 of experiments were also used in Round 2, barring mortality.

**Table 1:** Summary tables displaying the observed proportion of snails consumed by crayfish, unavailable for crayfish consumption, or dead by causes other than crayfish consumption.

**a.**	**consumed**		**unavailable**		**dead**	
**temperature**	**n**	**mean (SD)**	**n**	**mean (SD)**	**n**	**mean (SD)**
**15°C**	106	0.15 (0.17)	95	0.09 (0.16)	174	0.03 (0.07)
**20°C**	109	0.30 (0.25)	95	0.13 (0.16)	181	0.02 (0.06)
**25°C**	108	0.33 (0.27)	95	0.11 (0.15)	178	0.02 (0.06)
**30°C**	110	0.38 (0.26)	95	0.11 (0.14)	181	0.02 (0.05)
**35°C**	107	0.23 (0.27)	92	0.15 (0.18)	176	0.01 (0.04)
**b.**	**consumed**		**unavailable**		**dead**	
**status**	**n**	**mean (SD)**	**n**	**mean (SD)**	**n**	**mean (SD)**
**infected**	119	0.33 (0.25)	233	0.13 (0.17)	233	0.01 (0.04)
**uninfected**	421	0.27 (0.26)	239	0.11 (0.15)	657	0.02 (0.06)
**c.**	**consumed**		**unavailable**		**dead**	
**time**	**n**	**mean (SD)**	**n**	**mean (SD)**	**n**	**mean (SD)**
**12 hrs.**	86	0.31 (0.27)	75	0.10 (0.14)	142	0.02 (0.06)
**24 hrs.**	88	0.36 (0.31)	79	0.11 (0.14)	147	0.02 (0.06)
**36 hrs.**	87	0.23 (0.21)	79	0.13 (0.17)	144	0.02 (0.06)
**48 hrs.**	86	0.31 (0.28)	79	0.09 (0.13)	144	0.01 (0.04)
**60 hrs.**	95	0.19 (0.21)	80	0.13 (0.17)	155	0.03 (0.05)
**72 hrs.**	98	0.28 (0.24)	80	0.14 (0.20)	158	0.02 (0.07)
**d.**	**consumed**		**unavailable**		**dead**	
**species**	**n**	**mean (SD)**	**n**	**mean (SD)**	**n**	**mean (SD)**
**Bi. glabrata**	268	0.27 (0.24)	236	0.10 (0.16)	449	0.01 (0.04)
**Bu. truncatus**	272	0.29 (0.27)	236	0.14 (0.15)	441	0.03 (0.07)

Proportions are grouped by (a) temperature treatments, (b) infection status, (c) trial time blocks, and (d) snail species. Because not all data were used to inform consumption estimates, and separate control trials were conducted without crayfish to assess snail predator-avoidance behavior, sample sizes differ among data used to evaluate consumption, predator avoidance, and other mortality. Proportions are given as a mean ± SD.

### Analysis

We were interested in the influence of temperature, snail species, and snail infection status on the rate at which crayfish consumed snails. We assumed that crayfish body size (measured as weight in grams) would also influence consumption, because larger animals should consume more and may respond differently to temperature compared to smaller animals. Over the course of the experiment, we encountered instances when there were too many snails in a tank (i.e., more snails than the set density of 12). This may have occurred if observers overlooked a hiding snail. When this occurred, the trial in which too many snails were observed and the previous trial were removed from analyses, resulting in the exclusion of 52 data points out of a total of 960 (5%). In addition, 16 instances of crayfish molting (the trial the molt was observed, as well as the previous and following trials) were excluded from analyses, as crayfish are known to cease feeding during molting [84]. Control tanks did not have crayfish (n = 350 observations) and were used to estimate background snail mortality. Therefore, only data from experimental trials (n = 542) were included in analyses of crayfish consumption. All analyses were run in R version 4.3.1 (2023-06-16) [85].

We analyzed crayfish consumption rates using a generalized linear mixed model (GLMM) implemented in the “glmmTMB” package in R [85, 86]. As each tank was stocked with 12 snails at the start of each trial, we used a binomial likelihood with a logit link function such that for the number of snails eaten *y*_*i*_ in each trial *i*:

$$y_{i}∼Binomial(p_{i},N_{s,i}-N_{d,i})$$

$$logit(p_{i})=\alpha +\beta X_{i}+\epsilon_{c,i}+\epsilon_{r,i}+\epsilon_{cr,i}$$

where *p*_*i*_ as the expected probability of consumption per-snail, *N*_*s*,*i*_ = 12 is the initial snail density, *N*_*d*,*i*_ is the number of snails which died but were not consumed, *α* is an intercept, and *β* is the vector of coefficients corresponding to the matrix of predictors *X*_*i*_. We chose a binomial model with a logit link because it (1) intrinsically accounts for the fact that the response *y*_*i*_ is bounded between 0 and 12, and (2) accounts for the sigmoidal mean–variance relationship which is typical of binary outcome (e.g., consumed / not consumed) data; neither of these conditions are met by a Gaussian or Poisson likelihood [87]. As fixed effects, we fit snail species (2 levels), infection status (2 levels), temperature (5 levels: 15, 20, … 35°C), trial time (6 levels: 12, 24, … 72 hours), and all second- and third-order interactions among these predictors, as well as crayfish weight and the interaction between temperature and crayfish weight (Fig 1). To account for repeated observations of the same crayfish over several weeks of the experiment, we fit random intercepts for each crayfish (*ϵ*_*c*,*i*_) and experimental run (*ϵ*_*r*,*i*_), as well as an interaction between them to allow crayfish intercepts to vary across runs (*ϵ*_*cr*,*i*_). We used Wald *χ*^2^ tests to conduct null hypothesis tests of main effects and interactions at the *α* = 0.05 level [88]. Following significant main effects or interactions, we conducted post-hoc pairwise comparisons of the estimated marginal effects using the R package “emmeans”, adjusting p-values using the Tukey method [89], and using compact letters displays to aid interpretation [90].

In line with our experimental design, temperature and time were coded as categorical predictors, but the model estimates displayed an apparent unimodal effect of temperature and a negative, monotonic effect of time, so we conducted further tests to assess the continuous effects of these variables. For temperature, we fit a generalized additive mixed model (GAMM) using the R package “mgcv”, with the same response distribution and random effects structure as the above model, but including as fixed effects only a temperature spline and weight. We used this model to visualize continuous thermal consumption curves at 3, 7, and 11g (approximately the 10% quantile, mean, and 90% quantiles of observed crayfish weights, respectively). To assess the linear effect of time on per capita consumption rate implied by the estimates from the main model, we computed post-hoc polynomial contrasts, extracting the linear trend component and standard error following a multivariate normal distribution around the estimated marginal means and variance-covariance matrix (on the log-odds scale) for each time point [91, 92].

Though snail density was reset prior to the start of each trial, snails could have moved to various positions within the tank (i.e., above the water line, under the shelter/hiding) where they would be inaccessible to crayfish consumption during the trial. Anti-predatory behavior may be related to infection status, as infected snails are expected to move more slowly and less frequently than uninfected snails [60]. Therefore, we fit a model to assess the number of unavailable snails (the number above the water line or under shelter/hiding) as a function of snail species, snail infection status (uninfected or infected), and condition (control or experimental). As in the main model, we used a binomial likelihood and logit link and included all second- and third-order interactions among fixed effects. As crayfish were absent from the control trials, we used only random week effects in this model. We included only data from experimental round 2 (n = 472), as infected snails were not examined in round 1.

Lastly, we assessed whether snails died by causes other than predation in the control tanks, and whether there were differences in background mortality between the control and treatment tanks. We employed a binomial GLMM with a logit link, regressing the number of dead snails in each trial on the same set of fixed and random effects used for the unavailable snails model, but including data from both experimental runs (n = 890). For both models, we evaluated main effects and interactions using Wald tests and conducted Tukey-adjusted pairwise comparisons as above.

## Results

**Fig 2:**
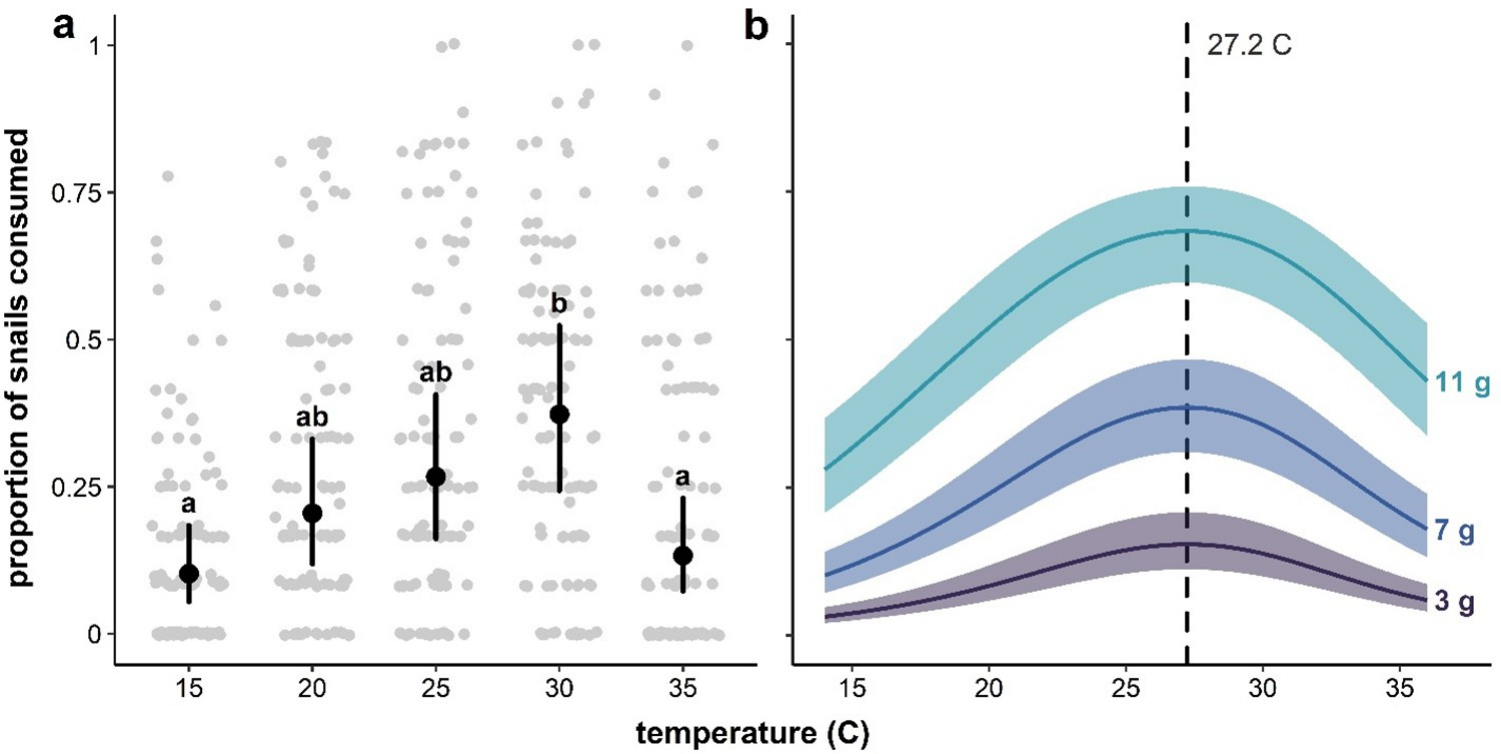
**(a)** Estimated marginal means and confidence intervals for probability of snail consumption at 15, 20, 25, 30, and 35°C, superimposed on jittered points corresponding to observed snail consumption proportions. (b) Continuous temperature curves (and 95% confidence bands) for 3, 7, and 11g individuals (approximately the 10% quantile, mean, and 90% quantile of crayfish weights). Estimates that do not share a letter are significantly different. Both discrete (GLMM estimated) and continuous (GAMM estimated) effects are consistent with a unimodal effect of temperature on consumption, with GAMM curves suggesting a peak around 27.2°C.

Snail consumption was significantly associated with temperature (*χ*^2^(4) = 29.1, p < 0.001), time point (*χ*^2^(5) = 97.1, p < 0.001), and crayfish weight (*χ*^2^(1) = 18.9, p < 0.001), but not with infection status (*χ*^2^(1) = 0.42, p = 0.52) or snail species (*χ*^2^(1) = 0.02, p = 0.88) (Fig 1). No second- or third-order interactions among the main effects were significant (S1 Table). Temperature displayed an apparent unimodal effect on consumption, with probability of consumption at the 30°C treatment (μ = 0.37, 95% CI = [0.24, 0.52]) significantly greater than at both the 15°C (μ = 0.10, 95% CI = [0.05, 0.18], p = 0.002) and 35°C treatments (μ = 0.13, 95% CI = [0.07, 0.23], p = 0.02, Fig 2). GAMM estimates suggest that consumption peaks between the 25 and 30°C treatments, at approximately 27.2°C.

**Fig 3:**
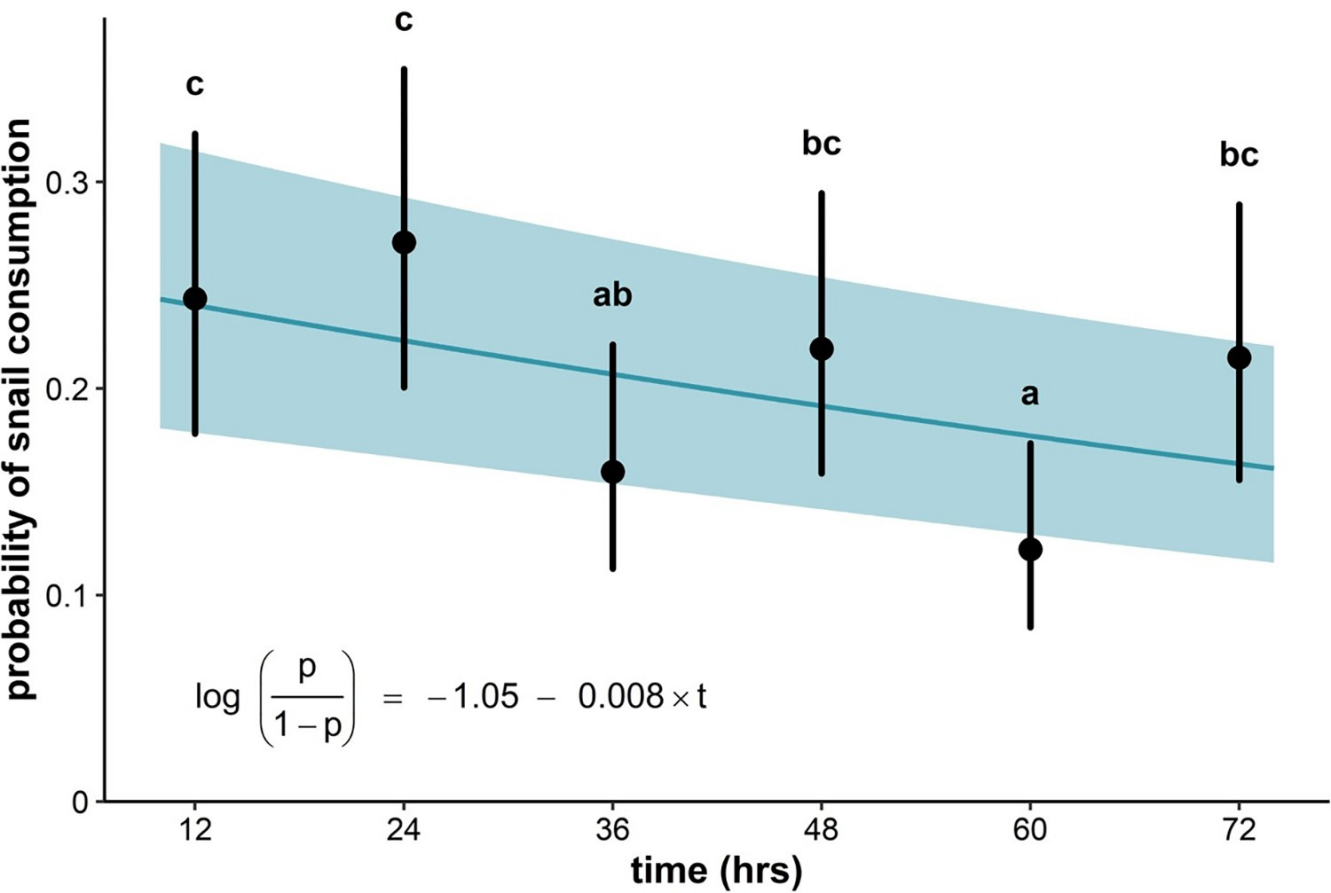
Estimated marginal means and confidence intervals for probability of snail consumption at trial time points (12 to 72 hrs at 12 hr intervals), superimposed on the lineartime trend (and 95% confidence band). Estimates that do not share a letter are significantly different. Results are consistent with a small but significant satiation effect. The linear trend is consistent with a ~9% decrease in the odds of snail consumption every 12 hours, indicative of a small but significant satiation effect (*e*^−0.008×12^ = 0.91, t(432) = -4.2, p < 0.001, r = - 0.49).

While larger crayfish consumed more snails across temperature treatments, the absence of a significant interaction between temperature and crayfish weight (*χ*^2^(4) = 1.02, p = 0.91) suggests that the shape and maxima of crayfish thermal consumption curves does not vary among crayfish of different sizes. Estimates of snail consumption probability across time points are consistent with a ~9% decrease in the odds snail consumption every 12 hours, indicative of a small but significant satiation effect (*e*^*β*×12^ = 0.91, t(432) = -4.2, p < 0.001, Fig 3).

**Fig 4:**
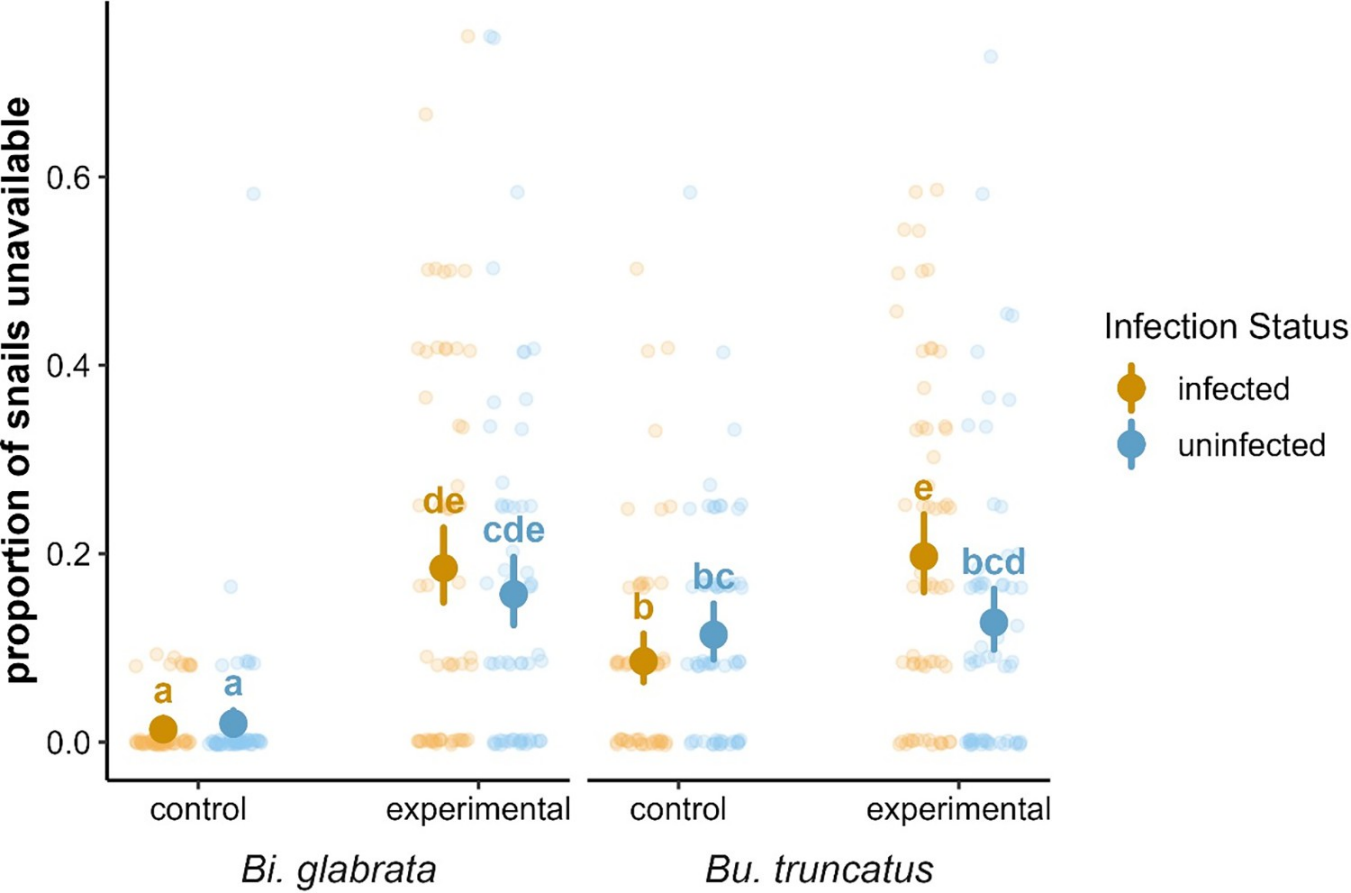
Estimated marginal means and confidence intervals for the proportion of unavailable snails across species, infection status, and experimental condition, superimposed on jittered points corresponding to observed proportions of unavailable snails in trials. Estimates that do not share a letter are significantly different.

The proportion of snails unavailable to prawns did not generally differ between infected and uninfected snails (*χ*^2^(1) = 3.5, p = 0.06, S2 Table). We did, however, find a significantly higher proportion of unavailable snails in experimental (μ = 0.17, 95% CI = [0.14, 0.20]) than in the control (μ = 0.04, 95% CI = [0.03, 0.05]) treatments (*χ*^2^(1) = 90.6, p < 0.001), supporting the hypothesis of a pro-active anti-predatory behavior. We also estimate significantly more unavailable *Bu*. *truncatus* (μ = 0.13, 95% CI = [0.10, 0.15])) than *Bi*. *glabrata* (μ = 0.06, 95% CI = [0.04, 0.07])) snails (*χ*^2^(1) = 9.1, p = 0.003). There was a significant interaction between experimental condition (control vs treatment) and species (*χ*^2^(1) = 66.14, p < 0.001), with more unavailable *Bu*. *truncatus* than *Bi*. *glabrata* in control tanks (Fig 4). There was also a significant interaction between condition and infection status (*χ*^2^(1) = 66.14, p < 0.001), which appears to be driven by a significantly higher proportion of infected (μ = 0.20, 95% CI = [0.16, 0.24]) than uninfected (μ = 0.13, 95% CI = [0.10, 0.16]) *Bu*. *truncatus* in the experimental tanks (p = 0.009) with no other significant differences between uninfected and infected snails (Fig 4).

Minimal snail mortality occurred in control conditions. Of 350 total 12-hour long control trials, there were only 20 instances (6% of trials) in which the number of snails remaining in the tank was less than the original number of 12. Specifically, mortality was 0.2% ± 0.1% (SE) for *Bu*. *truncatus* and 0.5% ± 0.1% for *Bi*. *glabrata* in control tanks. Conversely, mortality in experimental tanks was significantly higher than in control tanks both for *Bu*. *truncatus* (4.6% ±0.5%, Wald test t(881) = -5.17, p < 0.001, S3 Table) and for *Bi*. *glabrata* (1.7% ±0.3%, t(881) = -3.06, p = 0.012), corresponding to a nearly 50-fold reduction in projected life expectancy for *Bu*. *truncatus* and a nearly 7-times reduction in projected life expectancy for *Bi*. *glabrata* under laboratory predation conditions (S1 Fig).

## Discussion

In this set of experiments, the rate at which marbled crayfish consumed schistosome-competent snails was significantly influenced by temperature and crayfish weight. Per-capita crayfish consumption was shown to be a unimodal function of water temperature that peaked at intermediate temperatures between 26 and 29°C. Consumption, however, was not influenced by other factors, such as snail species and snail infection status and there was no interaction between temperature and weight. Crayfish consumed fewer snails at the low and high temperature extremes (15, 20, and 35°C, respectively) compared to moderate temperatures (25 and 30°C). In our experiment, 15°C and 20°C occurred on the rising slope of the crayfish’s thermal consumption curve and 35°C on the falling slope. Crayfish consumption generally increased with increasing temperature until ~27.5°C, at which point consumption declined. Though a similar trend emerges in previous studies, thermal optima, or the temperature at which the rate of consumption is maximized, varies considerably amongst crayfish species [74–76]. Taken together, our results demonstrate that, under laboratory conditions, marbled crayfish are voracious predators of schistosome-competent snails and that this regulatory effect is modulated by climatic conditions. These results provide a strong foundation for future investigation of the potential of marbled crayfish to regulate human disease burden by reducing intermediate host snails. Field studies are warranted to determine the regulatory effect of the invasive marbled crayfish on snail populations—and, in turn, human schistosomiasis burdens—across a broad thermal range, including habitats falling outside of the range of naturally occurring snail predators. Future studies should also investigate the relationship between marbled crayfish presence and human schistosomiasis burden, which cannot be explicitly examined within the scope of this work.

In general, larger crayfish consumed a greater number of snails across all temperatures. This pattern is consistent with previous findings for other crustacean species, in which large crayfish (*P*. *clarkii*) and prawns (*Macrobrachium* spp.) consumed significantly more schistosome-competent snails than smaller conspecifics [80, 93, 94]. However, marbled crayfish consumed fewer *Bi*. *glabrata* and *Bu*. *truncatus* snails, on average, than did prawns of similar size (extra small to medium-sized prawns, ranging from < 1 g to 10 g; consumption rate = 4.67 snails/12 hours).

Our results suggest that abiotic (temperature) and biotic (crayfish weight) factors influence rates of consumption and, in turn, the potential regulatory control of the marbled crayfish on snail host populations. However, interpretation of the present findings must be contextualized within the natural ecosystems in which *Schistosoma* transmission takes place, and therefore, we must bear in mind that crayfish are just one player impacted by temperature. Temperature plays an important, if not *the* most important, role in the distribution of schistosome-competent snails and human schistosomiasis at the large geographical scale [95, reviewed in 96, 97]. Snail hatching, growth, fecundity, and survivorship are also substantially impacted by temperature [81, 82 reviewed in 96, 97]. In an experiment investigating the influence of temperature on the biology of schistosome-competent snails, Sturrock and Sturrock [82] found that *Bi*. *glabrata* snails held at 25°C and 30°C hatched two weeks earlier than conspecifics held at 20°C. Egg hatching was not observed in 35°C aquaria, and hatchlings transferred to these tanks did not survive past two weeks. Following hatching, growth rate increased with increasing temperature (20–30°C), and snails in the 25°C and 30°C tanks reached sexual maturity and began laying eggs eight weeks earlier than snails in 20°C tanks. The number of eggs laid per two-week period varied considerably across temperature conditions, with snails laying six to eight times as many eggs in the 25°C condition compared to other conditions. Survivorship was also highest in 25°C tanks [82]. Likewise, temperature impacts the penetration and subsequent development of *Schistosoma* miracidia (measured as the number of daughter sporocysts) [98] in snail intermediate hosts, as well as the production of free-swimming cercariae [99], cercarial emergence [100], and survival [101]. Miracidial infection in snails increased with increasing temperature (ranging from 10°C to 40°C at 3°C intervals), as did the number of daughter sporocysts produced within a snail host to a point (production decreased at 40°C) [98]. Cercarial production increases between 15°C and 31°C [99]. As snail metabolic activity, energy, and vitality (e.g., fecundity, survival, and mortality rate) increases with rising temperatures (to a point), so does cercarial production with the snail intermediate host [100, 101, reviewed in 96]. In general, cercariae survival decreases as the water temperature increases, with exponential increases in mortality occurring at temperatures below 15°C and greater than 35°C [101]. Taken together, the results of our study demonstrate that the optimum temperature for crayfish consumption coincides well with that of snail egg production and hatching [82]. When crayfish consumption of intermediate snail hosts is expected to be hindered by low or high temperatures (i.e., the thermal limit), as demonstrated by the present study, cercarial production and survival–and, in turn, the risk of schistosomiasis transmission and infection–is also expected to be lower [99, 101].

An important finding was that neither snail species nor snail infection status were significantly associated with rates of marbled crayfish consumption. Swartz and colleagues [60] postulated that schistosomiasis control efforts may be enhanced by snail predators, if infected snails are consumed preferentially. On the other hand, intervention efforts may be hindered should predators avoid consuming infected snail hosts. Prawns (*Macrobrachium vollenhovenii*) preferentially consume *Bi*. *glabrata* snails exposed to *S*. *mansoni* and *Bu*. *truncatus* snails exposed to *S*. *haematobium* [60], but we found that marbled crayfish did not preferentially consume infected snails. One possible explanation is that *Schistosoma* spp. infections were not given adequate time to develop initially or that exposure to differing temperatures resulted in variations in the rate at which infections developed [102], making the difference between uninfected and infected snails negligible. While prawns are native to the sub-Saharan riverine system, the marbled crayfish evolved only recently [12–15] from a progenitor species (*Procambarus fallax*) that has a distribution that does not overlap with schistosome-infected snails [103, 104]. As such, marbled crayfish have not co-evolved with schistosome-competent snails, meaning that snails, regardless of infection status, may not have yet developed anti-predator behavior to this unfamiliar predator. Alternatively, the marbled crayfish, as a recently evolved invader, may also not be able to detect physiological differences between uninfected and infected snails that may otherwise make infected animals more detectable or desirable to predators [105]. Therefore, differences in predator preference between infected and uninfected snails may be diminished in the interaction between marbled crayfish and snails. The present study may have also lacked statistical power, hindering our ability to detect an effect of snail infection status on crayfish consumption rates across time points.

We postulated that crayfish consumption of uninfected and infected snails would be influenced by the number of snails available to be consumed, which could differ depending on snail infection status (as described above) [60]. We observed more unavailable snails in experimental trials compared to control trials, indicating that both *Bi*. *glabrata* and *Bu*. *truncatus* snails respond to the presence of marbled crayfish with enhanced anti-predator behavior. Snail availability, however, did not generally differ between infected and uninfected snails in either the experimental or control conditions. This suggests that both *Bu*. *truncatus* and *Bi*. *glabrata* snails, regardless of infection status, engage in anti-predator behaviors when exposed to a marbled crayfish individual in the experimental condition. The fact that there were no systematic differences in the number of unavailable snails between the infected and uninfected conditions suggests that the lack of an overall effect of infection status on crayfish consumption rates probably does not arise from opposing effects of preference and accessibility (i.e., our experiments do not support the hypothesis that crayfish prefer infected snails but infected snails are harder to get, or vice versa). These findings contrast those of Swartz and colleagues [60], who found that snails infected with *Schistosoma* spp. exhibit a diminished anti-predator response compared to uninfected snails when exposed to simulated predation cues–an unexpected outcome given that the two studies used genetically identical strains of snails and parasites. However, in the present experiment, snail behavior and crayfish consumption are confounded in the experimental treatment; that is, the difference between the number of unavailable snails in control versus experimental treatments could be due either to differences in snail behavior (i.e., more snails are choosing habitats that make them unavailable in the experimental condition compared to the control condition) or crayfish consumption (i.e., there are fewer unavailable snails because crayfish have eaten snails in the predation experiments). It is therefore possible that, in the absence of crayfish consumption (by caging crayfish in the experimental tanks and preventing them to predate on snails), we might have observed a greater number of unavailable snails in the experimental treatment, and this would have revealed more marked expression of anti-predator behavior among uninfected snails compared to infected snails, consistent with Swartz et al. [60]. Future studies should monitor uninfected and infected snail behavior in a simulated predator condition (described by [60]), where the marbled crayfish is present but unable to access and consume the snails and consumption is simulated through the addition of crushed conspecifics.

As with any experimental study, several caveats are worth noting. Behavioral differences are known to arise between aquarium- and naturally reared marbled crayfish; aquarium-reared individuals tend to be more active and aggressive [105], suggesting that laboratory consumption rates may not reflect the rates occurring in nature. However, previous field observations in Madagascar found that native snails were absent from areas in which the marbled crayfish had established [65], providing supportive (although not conclusive) field evidence that this invader may prey upon snails in nature. Previous studies have also demonstrated links between the abundance of schistosome-competent snails and human infection burdens [38, 42, 60, 80, 106]. Though this is promising evidence in support of the crayfish’s potential to serve as a biological control of snail intermediate hosts, our results demonstrate that ecological parameters, such as crayfish weight and temperature, could influence the relationship between invasive enemies, snails, and human schistosomiasis burden. For example, based on peak rates of consumption from the experiment, crayfish in lower (<25°C) and higher (>30°C) water temperatures may be less likely to reduce snail populations. Anyway, no sustained transmission of schistosomiasis seems to occur when average temperature in the warmest quarter exceeds ca. 31–32 C [97], so other ecological processes concur to limit transmission risk at high temperature and reduction of crayfish consumption rate in the upper thermal range of the distribution won’t be relevant. On the other hand, per-capita crayfish consumption rate might be significantly lower than at peak in the Madagascar highland where mean annual temperature is around 25C or below. Also, our experiments showed that per capita consumption rate in the 6 consecutive 12-hour long trials of the predation experiments slightly decreased with time, a possible indication of satiation, thus slightly reducing the effectiveness of predation control by marbled crayfish. Jointly with the observation that snails exhibited a clear anti-predatory behavior and actively searched for predation refugia, there is the possibility that in natural wildlife conditions, predation rate might be lower than estimated in our laboratory experiments.

While invasion of alien species should be avoided by any means, we observe that, according to the results of this laboratory experiment and field evidence of a reduction of snail abundance where the crayfish invaded, the presence of the marbled crayfish may provide an unexpected co-benefit to the people of Madagascar: crayfish-driven reductions in burdens of human schistosomiasis [38, 41, 42]–something that is urgently needed in a country where prevalence in some villages can range up to 94% [66, 67]. However, this effect needs to be weighed against other considerations as the country leadership grapples with all the ecological and social impacts of a significant number of invasive species [107] and, specifically, of the marbled crayfish. Several studies have clearly documented that the invasion of the Louisiana crayfish *Procambarus clarkii* can have dramatic impacts on native invertebrate fauna [108, 109]. Introduced marbled crayfish directly threaten Madagascar’s native aquatic wildlife [65]–a pressing concern given approximately 90% of plant species, 36% of birds, 90% of mammals, 96% of reptiles, 33% of fish and 86% of macroinvertebrates are endemic to the island [110].

On the other hand, natural predators of the obligate host snail of schistosome parasites may be playing an important role in the diet of children [111, 112], serving as an opportunistic and rapidly renewable nutritional source in a country where ~50% of children experience stunted growth due to lack of sufficient dietary protein [113]. Non-native species have long been recognized as having profound effects on ecosystem services [7–9] through their influence on the abundance and distribution of their prey and competitors [10, reviewed in 11]. However, empirical evidence of such consequences does not exist for the vast majority of non-native introductions [114–116]. Non-native species may, in some regards, have negligible and even positive impacts on ecosystems [43, 117], and careful, balanced evaluations of all the benefits and disservices associated with species introductions are needed to better inform management strategies [43, 117–119]. Drawing upon examples of non-native crayfish, the impact of a non-native species on ecosystems may vary considerably and is probably species-specific and context-dependent [120–123]—thus limiting our ability to make broad predictions regarding the multi-faceted impacts of invasive species on biodiversity and ecosystem services, including the regulation of disease agents. In general, existing evidence of the positive impact of natural enemies on human diseases is exceedingly rare, as are empirical investigations linking species interactions within the environment to outcomes of human disease [reviewed in 2].

Our study shows unequivocally that crayfish are voracious predators of schistosome-competent snails and that temperature modulates consumption rates. Whether crayfish are able to reduce schistosomiasis prevalence in the human population by controlling snail abundance at the transmission sites has yet to be determined. Field studies are needed to determine whether the snail consumption documented here will translate into impacts on snail populations and human schistosomiasis burden. However, the possibility remains that reductions in schistosomiasis transmission might be a silver lining to the invasion of *Procambarus virginalis* in Madagascar. Before any proactive attempt to deploy non-native species for schistosomiasis control, future studies will need to carefully quantify the co-occurring ecological impacts and potential benefits for nutrition and reduction in schistosomiasis transmission risk of nature-based solutions for disease control. Until then, it is imperative that the invasive crayfish must not be introduced to freshwaters beyond its current range. The present work lays the foundation for future exploration of the potential role of marbled crayfish in the regulation of schistosome-competent snails and draws attention to the untapped potential for non-native species to regulate disease.

## Supporting information

S1 Fig Estimated means and confidence intervals for snail mortality across species experimental condition. Estimates that do not share a letter are significantly different.
(TIF)

S1 Table Wald tests for main effects and interactions for binomial GLMM fit to the number of consumed snails in experimental trials. Asterisks indicate significant predictors.
(DOCX)

S2 Table Wald tests for main effects and interactions for binomial GLMM fit to the number of unavailable snails in control and experimental trials. Asterisks indicate significant predictors.
(DOCX)

S3 Table Wald tests for main effects and interactions for binomial GLMM fit to the number of dead snails in control and experimental trials. Asterisks indicate significant predictors.
(DOCX)
