# Supplementary figures and images for: Temperature affects predation of schistosome-competent snails by a novel invader, the marbled crayfish Procambarus virginalis
Source: PLoS One. 2023 Sep 13;18(9):e0290615. doi: 10.1371/journal.pone.0290615 (PMC10499222; doi:10.1371/journal.pone.0290615)

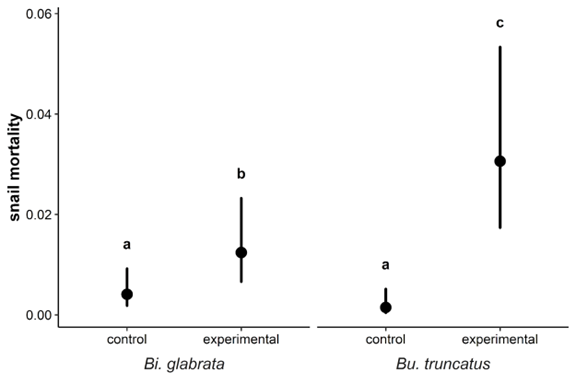

Supplement: S1 Fig — Estimates that do not share a letter are significantly different. (TIF) [file pone.0290615.s001.tif]
