# Supplementary material for: Temperature affects predation of schistosome-competent snails by a novel invader, the marbled crayfish Procambarus virginalis
Source: PLoS One. 2023 Sep 13;18(9):e0290615. doi: 10.1371/journal.pone.0290615 (PMC10499222; doi:10.1371/journal.pone.0290615)
Supplement: S1 Table — Asterisks indicate significant predictors. (DOCX) [file pone.0290615.s002.docx]

**Table S1.** Wald tests for main effects and interactions for binomial GLMM fit to the number of consumed snails in experimental trials. Asterisks indicate significant predictors.

| term | $\boldsymbol{\chi}^{\boldsymbol{2}}$ | df | Pr(>$\boldsymbol{\chi}^{\boldsymbol{2}}$) |  |
| --- | --- | --- | --- | --- |
| snail species | 0.023 | 1 | 0.881 |  |
| infection status | 0.422 | 1 | 0.516 |  |
| temperature | 29.056 | 4 | <0.001 | * |
| time | 97.1 | 5 | <0.001 | * |
| weight | 18.948 | 1 | <0.001 | * |
| snail species : infection status | 0 | 1 | 0.995 |  |
| snail species : temperature | 3.098 | 4 | 0.542 |  |
| snail species : time | 7.244 | 5 | 0.203 |  |
| infection status : temperature | 2.059 | 4 | 0.725 |  |
| infection status : time | 3.831 | 5 | 0.574 |  |
| temperature : time | 16.228 | 20 | 0.702 |  |
| temperature : weight | 1.021 | 4 | 0.907 |  |
| snail species : infection status : temperature | 2.706 | 4 | 0.608 |  |
| snail species : infection status : time | 10.049 | 5 | 0.074 |  |
| snail species : temperature : time | 20.826 | 20 | 0.407 |  |
| infection status : temperature : time | 19.352 | 20 | 0.499 |  |
