# Supplementary material for: Temperature affects predation of schistosome-competent snails by a novel invader, the marbled crayfish Procambarus virginalis
Source: PLoS One. 2023 Sep 13;18(9):e0290615. doi: 10.1371/journal.pone.0290615 (PMC10499222; doi:10.1371/journal.pone.0290615)
Supplement: S2 Table — Asterisks indicate significant predictors. (DOCX) [file pone.0290615.s003.docx]

**Table S2.** Wald tests for main effects and interactions for binomial GLMM fit to the number of unavailable snails in control and experimental trials. Asterisks indicate significant predictors.

| term | $\boldsymbol{\chi}^{\boldsymbol{2}}$ | df | Pr(>$\boldsymbol{\chi}^{\boldsymbol{2}}$) |  |
| --- | --- | --- | --- | --- |
| snail species | 9.103 | 1 | 0.003 | * |
| infection status | 3.526 | 1 | 0.06 |  |
| condition (control vs. experimental) | 90.575 | 1 | <0.001 | * |
| snail species : infection status | 2.318 | 1 | 0.128 |  |
| snail species : condition | 66.375 | 1 | <0.001 | * |
| infection status : condition | 14.679 | 1 | <0.001 | * |
| snail species : infection status : condition | 0.28 | 1 | 0.597 |  |
